# PHLDA1 Mediates Drug Resistance in Receptor Tyrosine Kinase-Driven Cancer

**DOI:** 10.1016/j.celrep.2018.02.028

**Published:** 2018-02-28

**Authors:** Abbie E. Fearon, Edward P. Carter, Natasha S. Clayton, Edmund H. Wilkes, Ann-Marie Baker, Ekaterina Kapitonova, Bakhouche A. Bakhouche, Yasmine Tanner, Jun Wang, Emanuela Gadaleta, Claude Chelala, Kate M. Moore, John F. Marshall, Juliette Chupin, Peter Schmid, J. Louise Jones, Michelle Lockley, Pedro R. Cutillas, Richard P. Grose

**Affiliations:** 1 Centre for Tumour Biology, Barts Cancer Institute—a CRUK Centre of Excellence, Queen Mary University of London, London EC1M 6BQ, UK; 2 Integrative Cell Signalling and Proteomics, Centre for Haemato-Oncology, Barts Cancer Institute, London EC1M 6BQ, UK; 3 Centre for Molecular Oncology, Barts Cancer Institute, London EC1M 6BQ, UK; 4 Centre for Experimental Cancer Medicine, Barts Cancer Institute, London EC1M 6BQ, UK

**Keywords:** tyrosine kinase inhibitor, drug resistance, FGF, Akt, targeted therapy, cancer

## Abstract

Development of resistance causes failure of drugs targeting receptor tyrosine kinase (RTK) networks and represents a critical challenge for precision medicine. Here, we show that PHLDA1 downregulation is critical to acquisition and maintenance of drug resistance in RTK-driven cancer. Using fibroblast growth factor receptor (FGFR) inhibition in endometrial cancer cells, we identify an Akt-driven compensatory mechanism underpinned by downregulation of PHLDA1. We demonstrate broad clinical relevance of our findings, showing that PHLDA1 downregulation also occurs in response to RTK-targeted therapy in breast and renal cancer patients, as well as following trastuzumab treatment in HER2^+^ breast cancer cells. Crucially, knockdown of PHLDA1 alone was sufficient to confer *de novo* resistance to RTK inhibitors and induction of PHLDA1 expression re-sensitized drug-resistant cancer cells to targeted therapies, identifying PHLDA1 as a biomarker for drug response and highlighting the potential of PHLDA1 reactivation as a means of circumventing drug resistance.

## Highlights

• The Akt pathway plays a critical role in resistance to tyrosine kinase inhibition
• PHLDA1 is key to regulating Akt activity during the development of drug resistance
• Knockdown of PHLDA1 alone confers *de novo* resistance to kinase inhibitors
• PHLDA1 rescue resensitizes drug-resistant cancer cells

## Graphical Abstract

**Figure:**
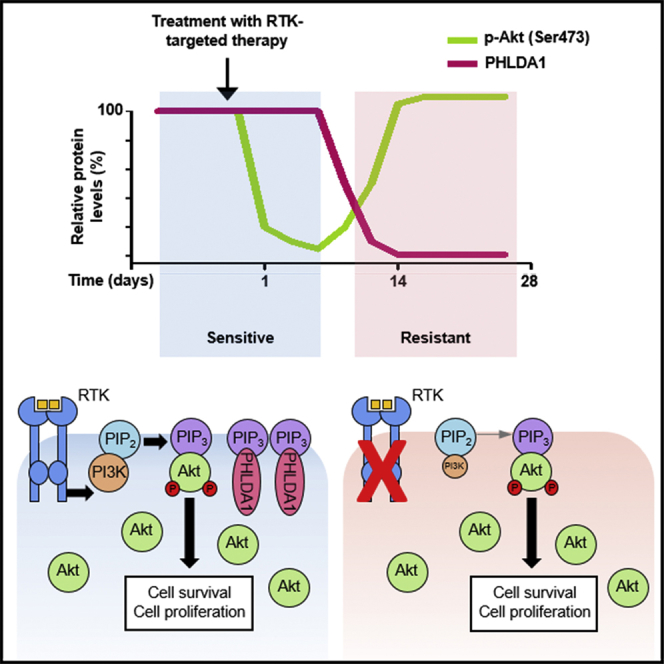

## Teaser

Fearon et al. use unbiased transcriptomic and phosphoproteomic analysis to identify PHLDA1 as a mediator of acquired resistance to kinase-targeted therapies in cancer. Using a range of cell models and clinical data, they uncover a mechanism underpinning the re-wiring of Akt signaling in cancer drug resistance.

## Introduction

Improved understanding of the molecular mechanisms underpinning cancer has led to the development of an arsenal of therapeutics, with which to tackle cancers driven by specific pathways. In particular, receptor tyrosine kinases (RTKs) have been implicated in a wide variety of oncogenic behaviors, driving cell proliferation, survival, migration, and mediating cancer cell-stromal crosstalk (Gross et al., 2015). Small molecule kinase inhibitors and therapeutic antibodies targeted to RTKs can offer clinically significant patient benefit (Slamon et al., 2001, Geyer et al., 2006). However, the selective pressure applied on cancer cells by targeting RTKs results in rapid evolution of resistance mechanisms, reducing the efficacy of targeted approaches and resulting in tumor re-growth in patients (Engelman et al., 2007, Kobayashi et al., 2005). Overcoming this acquired resistance to targeted therapies represents a critical challenge for cancer research (Holohan et al., 2013).

Fibroblast growth factor receptor (FGFR) signaling has been implicated in both oncogenic and drug-resistance mechanisms in many different cancers, and FGFR inhibitors are currently in clinical trials for a range of cancer types (Carter et al., 2015). Among these, endometrial cancer is ideal for exploring mechanisms of resistance. The fourth most common form of cancer in women, it highlights some very well-characterized FGFR driver mutations, with up to 16% of cases driven by mutations in FGFR2 (Fearon et al., 2013, Pollock et al., 2007). Herein, using 2D and 3D cultures of endometrial cancer cell lines expressing either wild-type or mutant FGFR2 as model systems, we have interrogated the mechanisms of acquired resistance to FGFR-targeted ATP mimetic small molecule therapies (Mohammadi et al., 1998, Gavine et al., 2012). Through gene expression profiling, we have identified loss of the protein Pleckstrin Homology-Like Domain, family A, member 1 (PHLDA1) as a critical mediator of resistance to FGFR inhibition and validated these studies by manipulating PHLDA1 expression. Using phosphoproteomics and combination drug treatment, we show that Akt signaling is critical to this acquired resistance, and we present a model for how PHLDA1 may mediate this effect. Finally, we have extrapolated our findings to other RTK-driven cancers, using cell-based, *in vivo* and bioinformatics approaches, to identify PHLDA1 as a mediator of resistance with direct relevance to a broad range of RTK-targeted therapies.

## Results

### Development of Drug Resistance in Endometrial Cancer Cells

**Figure 1:**
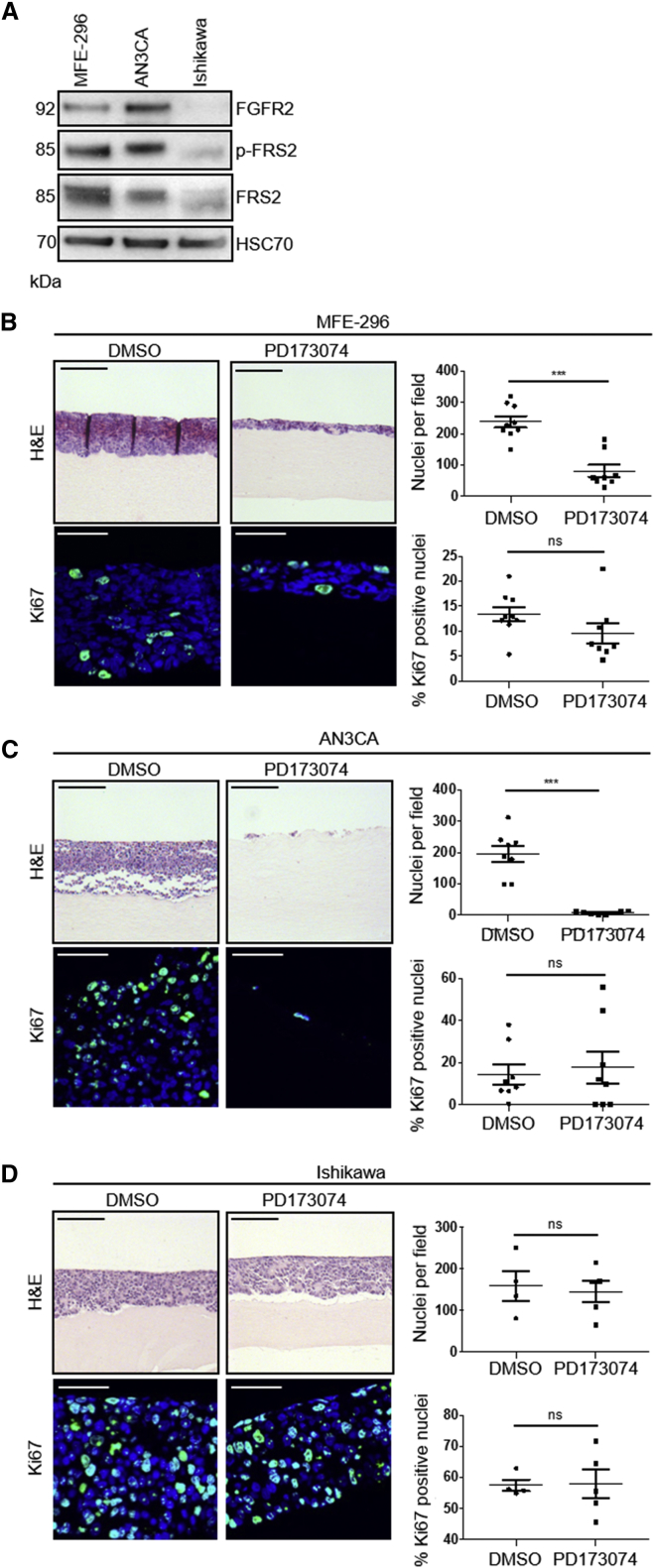
Generation of FGFR Inhibitor-Resistant Endometrial Cancer Cell Populations *In Vitro* (A) Western blot analysis of FGFR2 and phosphorylated FRS2α (Tyr436) in serum-starved MFE-296, AN3CA, and Ishikawa cells. Data are representative of three independent experiments. (B–D) Upper: H&E staining of MFE-296 (B), AN3CA (C), and Ishikawa (D) cells grown in organotypic cultures for 14 days with or without 1 μM PD173074. Lower: Ki67 staining with nuclei counterstained by DAPI. Right: quantitation of cell number and Ki67 positive nuclei per field of view. Data are presented as mean ± SEM. Images are representative of at least three independent experiments. H&E image scale bar, 100 μm; Ki67 image scale bar, 50 μm. ^∗∗∗^p ≤ 0.001, compared with DMSO controls. H&E images are automatically spliced composites.

To investigate mechanisms of acquired resistance to FGFR inhibitors, we adopted endometrial cancer cell line models, with two cell lines that harbor FGFR2 activating mutations, MFE-296 and AN3CA cells (Byron et al., 2008), and one that expresses wild-type FGFR2, Ishikawa cells (Byron et al., 2013). MFE-296 and AN3CA cells expressed high levels of FGFR2, relative to Ishikawa cells, and exhibited enhanced levels of phosphorylated FGFR substrate 2 (FRS2), an indicator of FGFR activation, reflecting their dependence on basal FGFR activation (Figure 1A). Ishikawa cells express wild-type FGFR and thus have minimal phosphorylated FRS2 under normal conditions.

To model FGFR inhibition in a physiologically relevant context, where cancer cells receive stromal support, 3D organotypic models (Chioni et al., 2017) were used (Figure 1). Collagen/Matrigel gels were overlaid with MFE-296, AN3CA, or Ishikawa cells and treated with FGFR inhibitors for 14 days. Treatment with PD173074, an ATP-competitive inhibitor of FGFR (Mohammadi et al., 1998), resulted in a significant reduction in cell number in MFE-296 and AN3CA cells, while Ishikawa cells remained unaffected (Figures 1B–1D). Quantitation of cell number and the percentage of Ki67-expressing cells following treatment revealed that AN3CA cells were almost absent following treatment with PD173074 (Figure 1C). In contrast, by day 14 of treatment, MFE-296 cells established a population of proliferating cells in the presence of PD173074, albeit reduced compared to DMSO control cultures (Figure 1B), suggesting the emergence of a resistant cell population. Comparable data were obtained using AZD4547, another ATP-competitive inhibitor of FGFR currently in clinical trials for FGFR2 mutant solid tumors (Carter et al., 2015, Gavine et al., 2012) (Figure S1).

To investigate the mechanism underlying sustained FGFR inhibitor resistance, FGFR-inhibitor-resistant populations of MFE-296 and AN3CA cells (MFE-296^AZDR^ and AN3CA^AZDR^, respectively) were generated by increasing exposure to AZD4547. When cultured on mini-organotypic gels for 7 days in the presence of 1 μM AZD4547, both populations of resistant cells showed significantly less reduction in proliferation rate compared to parental cells (Figures S1A and S1B). Drug sensitivities were confirmed in 2D culture using an IncuCyte platform and demonstrate that AN3CA cells exhibit enhanced sensitivity to both PD173074 and AZD4547 over MFE-296 cells, as previously reported (Packer et al., 2017) (Figures S1C–S1F). Further, both AN3CA^AZDR^ and MFE-296^AZDR^ exhibited cross-resistance to PD173074 (Figures S1D and S1F). As expected, Ishikawa cell growth was unaffected by both FGFR inhibitors (Figure S1G)

### Phosphoproteomic Interrogation of Resistance Acquisition in MFE-296 Cells

To understand how resistant cells are able to re-engage proliferative pathways, we examined changes to the phosphoproteome of MFE-296 cells, as they developed resistance to PD173074 over 14 days of culture.

**Figure 2:**
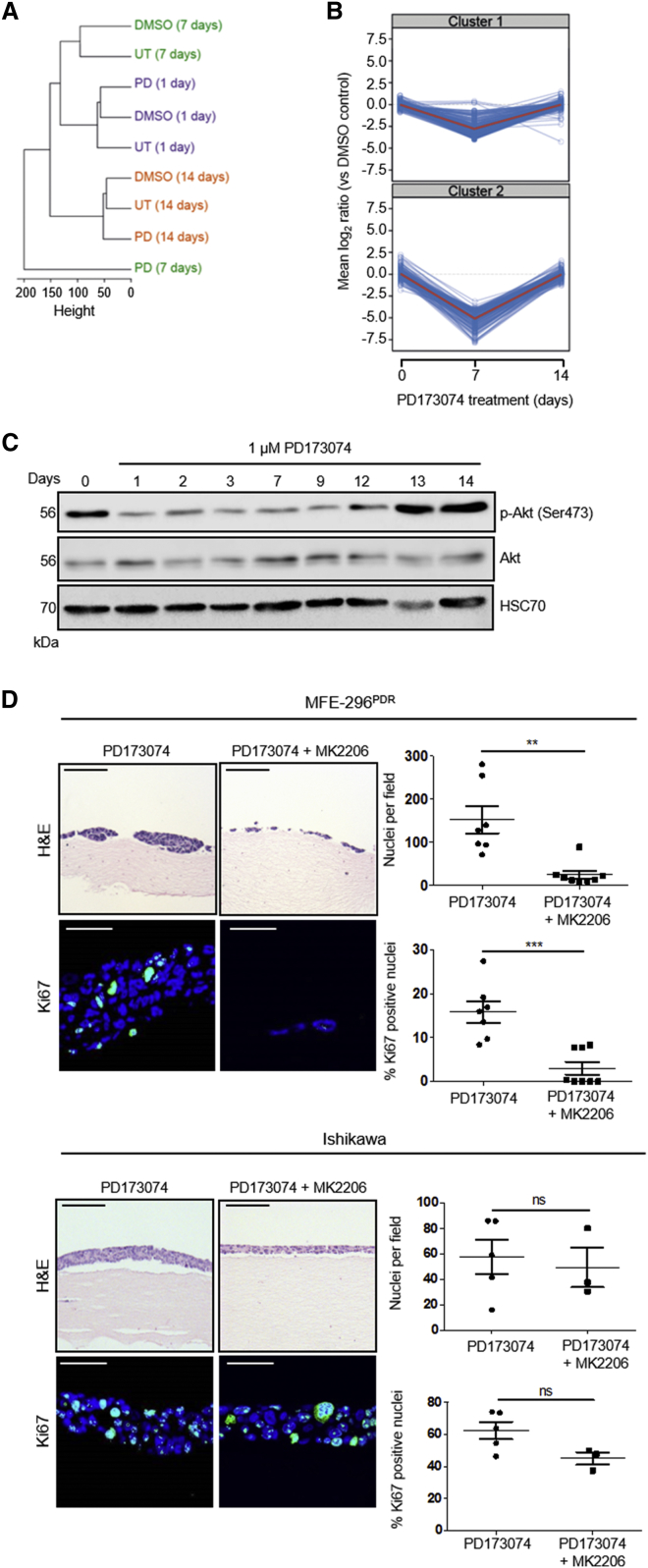
Phosphoproteomic Analysis of Endometrial Cancer Cell Lines Identifies a Pivotal Role for Akt Signaling in FGFR Inhibitor Resistance (A) Dendrogram of the hierarchical clustering (Pearson correlation distance metric) of phosphoproteomic signatures obtained through mass spectrometry of MFE-296 cells treated with DMSO, 1 μM PD173074 (PD), or untreated (UT) over 1, 7, and 14 days. (B) Representation of changes in the phosphoproteome of MFE-296 cells treated with 1 μM PD173074 compared to DMSO controls at 1, 7, and 14 days. (C) Western blot showing changes in pAkt (Ser473) induced by treatment of MFE-296 cells with 1 μM PD173074 over 14 days. Data are representative of three independent experiments. (D) Left: H&E staining and Ki67 staining of MFE-296^PDR^ cells (upper) and Ishikawa cells (lower) grown in organotypic cultures for 7 days. Cells were cultured in 1 μM PD173074 with or without 1 μM MK2206. Right: quantitation of cell number and Ki67 positive nuclei. Data are presented as mean ± SEM. Images are representative of at least three independent experiments. H&E images scale bar, 100 μm; Ki67 images scale bar, 50 μm. ^∗∗∗^p ≤ 0.001, ^∗∗^p ≤ 0.01. H&E images are automatically spliced composites.

Phosphorylation sites were identified and quantified using a well-established, label-free methodology (Alcolea et al., 2012, Casado et al., 2013, Wilkes et al., 2015). Hierarchical clustering of the resulting phosphorylation motifs demonstrated a high degree of similarity in the phosphoproteome between the start of treatment and that after 14 days of culture with PD173074. Intriguingly, the phosphoproteome of PD173074-treated cells was noticeably divergent from that of controls at 7 days of treatment, suggesting a global change in cell signaling (Figure 2A).

Of the 6,706 phosphopeptides identified, 525 were significantly up- or downregulated in samples from cells treated with PD173074, compared to DMSO controls, for at least one time point. These phosphopeptides were grouped according to their temporal profile (Figure S2A). Interestingly, 412 phosphopeptides were downregulated at 7 days treatment with PD173074 and returned to baseline levels after 14 days (Figure 2B).

Kinase substrate enrichment analysis (KSEA) (Casado et al., 2013) was used to determine the upstream kinases of the identified phosphopeptides. This approach identified an enrichment of Akt and Akt-related effectors (Figure S2A), indicating re-establishment of Akt signaling following an initial FGFR inhibitor-induced dampening of the pathway (Figures S2A and S2B). Supporting this, western blotting of cell lysates isolated from MFE-296 cells treated with PD173074 for up to 14 days confirmed that, while total Akt levels remained constant, there was a clear decrease in the levels of pAkt (Ser473), an indicator of Akt activity, which returned to normal within 13 days of continued treatment (Figure 2C).

To investigate the importance of Akt signaling in resistance, MFE-296^PDR^ cells were treated for 14 days with 1 μM PD173074, either alone or in combination with 1 μM MK2206, an allosteric pan-Akt inhibitor (Hirai et al., 2010), in organotypic models. This combination treatment reduced cell number and proliferation significantly, compared to single treatment alone (Figure 2D). In contrast, Ishikawa cells (FGFR2 wild-type) were unaffected by drug combination treatment (Figure 2D), indicating that the effects of PD173074/MK2206 treatment seen in MFE-296 cells were FGFR2 dependent. These data suggest that resurgence of Akt signaling mediates resistance to FGFR inhibition.

### Determining the Mechanism of FGFR Inhibitor Resistance in MFE-296 Cells

**Figure 3:**
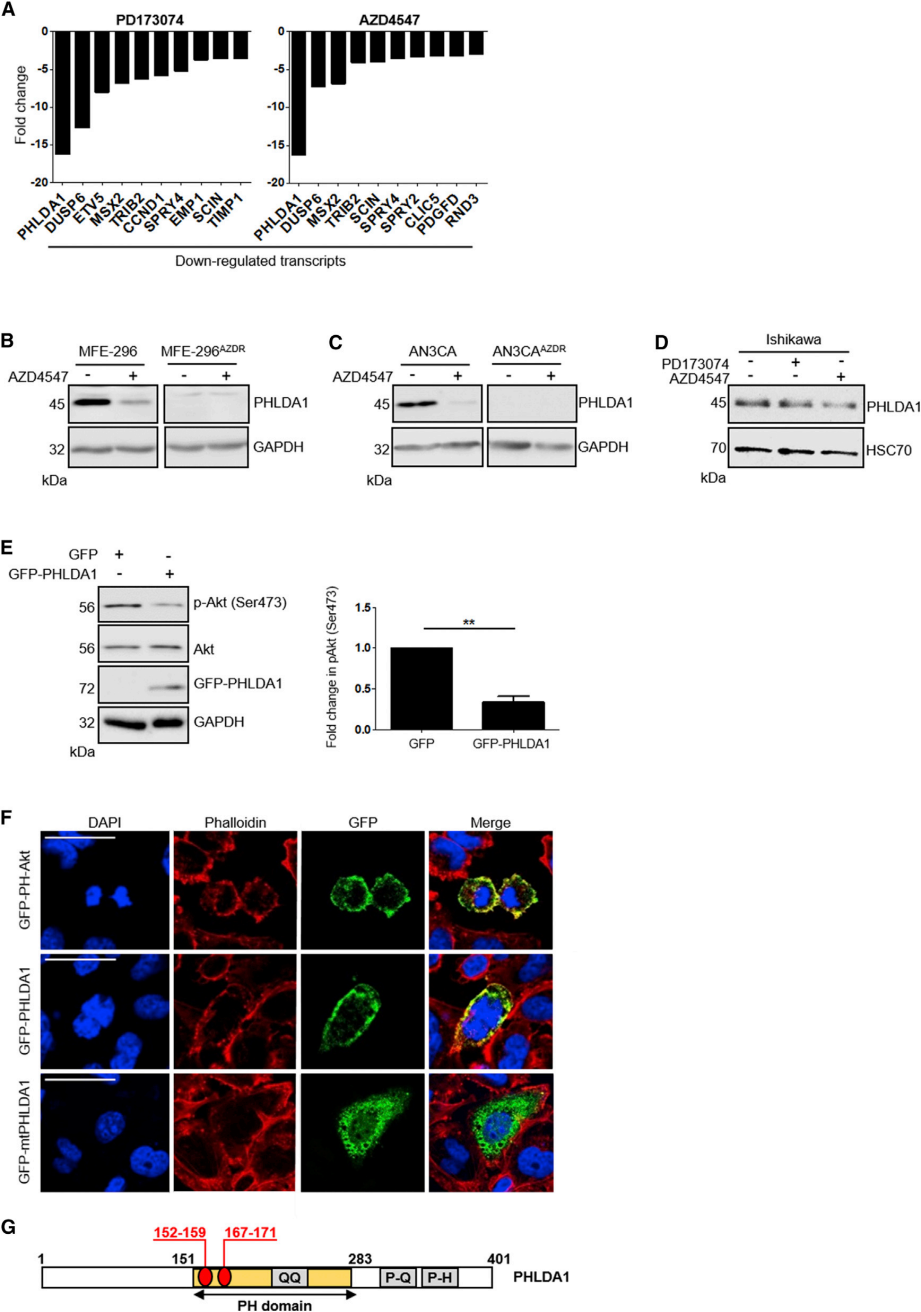
PHLDA1 Negatively Regulates Akt and Is Downregulated in FGFR Inhibitor-Resistant Endometrial Cancer Cell Lines (A) Top ten downregulated genes in MFE-296^PDR^ cells (left) and MFE-296^AZDR^ cells (right) compared to parental controls, identified by microarray analysis. (B–D) Western blot showing downregulation of PHLDA1 levels in parental MFE-296 (B) and AN3CA (C) cells following treatment with 1 μM AZD4547 for 24 hr and persistent downregulation of PHLDA1 in MFE-296^AZDR^ and AN3CA^AZDR^ cells following removal of 1 μM AZD4547 for 24 hr. PHLDA1 levels in Ishikawa cells (D) were unaffected by FGFR inhibitor treatment. (E) Left: western blot showing reduced p-Akt (pSer473) in HCC1954 cells following transfection with GFP-PHLDA1. Right: quantitation of p-Akt (Ser473), normalized to total Akt and GAPDH. Data are presented as mean fold change ±SEM in p-Akt (Ser473) ^∗∗∗^p ≤ 0.001. (F) MFE-296 cells were transfected with constructs encoding GFP-PHLDA1, GFP-mtPHLDA1, or GFP-PH-Akt for 48 hr prior to fixation. Nuclei were labeled with DAPI, and F-actin was visualized using Alexa Fluor 546 Phalloidin (red). Scale bar, 50 μm. (G) Domain organization of PHLDA1. PH domain, pleckstrin homology domain; QQ, polyglutamine tract; P-Q, proline-glutamine rich tract; P-H, proline-histidine rich tract. Residues deleted in mtPHLDA1 are indicated in red.

To investigate gene expression changes associated with FGFR inhibitor resistance, MFE-296, MFE-296^AZDR^ and MFE-296^PDR^ cells were assayed in duplicate using an Illumina platform. We identified 587 probes, corresponding to 522 genes, that were upregulated and 543 probes, corresponding to 485 genes, that were downregulated in MFE-296^PDR^ cells compared to the parental cell line. Among the top ten downregulated genes (Figure 3A) were several known FGFR signaling targets, including *Sprouty 4* (*SPRY4*) and *Dual specificity phosphatase 6* (*DUSP6*) (Li et al., 2007, Yang et al., 2006). Similarly, among the upregulated genes (Figure S3A), *IGFBP5* was identified, the expression of which is known to be elevated in the absence of FGFR2 in keratinocytes *in vivo* (Grose et al., 2007, Schlake, 2005). Interestingly, MFE-296^PDR^ and MFE-296^AZDR^ cells displayed strikingly similar changes in gene expression profile (Figures 3A, S3A, and S3B). The gene most significantly downregulated in both cell sub-populations was *PHLDA1* (Figure 3A).

PHLDA1 protein levels were decreased significantly in parental MFE-296 cells upon treatment with 1 μM AZD4547 or PD173074 for 7 days, and PHLDA1 protein was absent from MFE-296^AZDR^ and MFE-296^PDR^ cells, even following culture in drug-free medium (Figures 3B and S3C). These data were recapitulated in AN3CA and AN3CA^AZDR^ cells (Figure 3C), suggesting that stable downregulation of PHLDA1 levels is a common response to FGFR inhibition in these FGFR2-driven cancer cell lines. In line with this, PHLDA1 levels were unaffected in FGFR2 wild-type Ishikawa cells following PD173074 treatment (Figure 3D).

We next sought to determine whether PHLDA1 could regulate the activity of Akt, as has been previously implicated (Durbas et al., 2016, Li et al., 2014), thus providing a link between our proteomic and microarray datasets. Expression of a GFP-tagged PHLDA1 construct in the breast cancer cell line HCC1954 reduced the levels of pAkt (S473), suggesting negative regulation of Akt activation (Figure 3E). We also generated a mutant PHLDA1 construct wherein amino acid residues 152–159 and 167–171, corresponding to the predicted sites required for phosphatidyl-3, 4, 5-trisphosphate (PIP_3_) binding (Kawase et al., 2009), have been removed. This construct failed to localize to the cell membrane, unlike the wild-type counterpart, suggesting a requirement of a functional PH domain in the function of PHLDA1 (Figures 3F and 3G).

### Knockdown of PHLDA1 Confers Resistance to FGFR Inhibition

**Figure 4:**
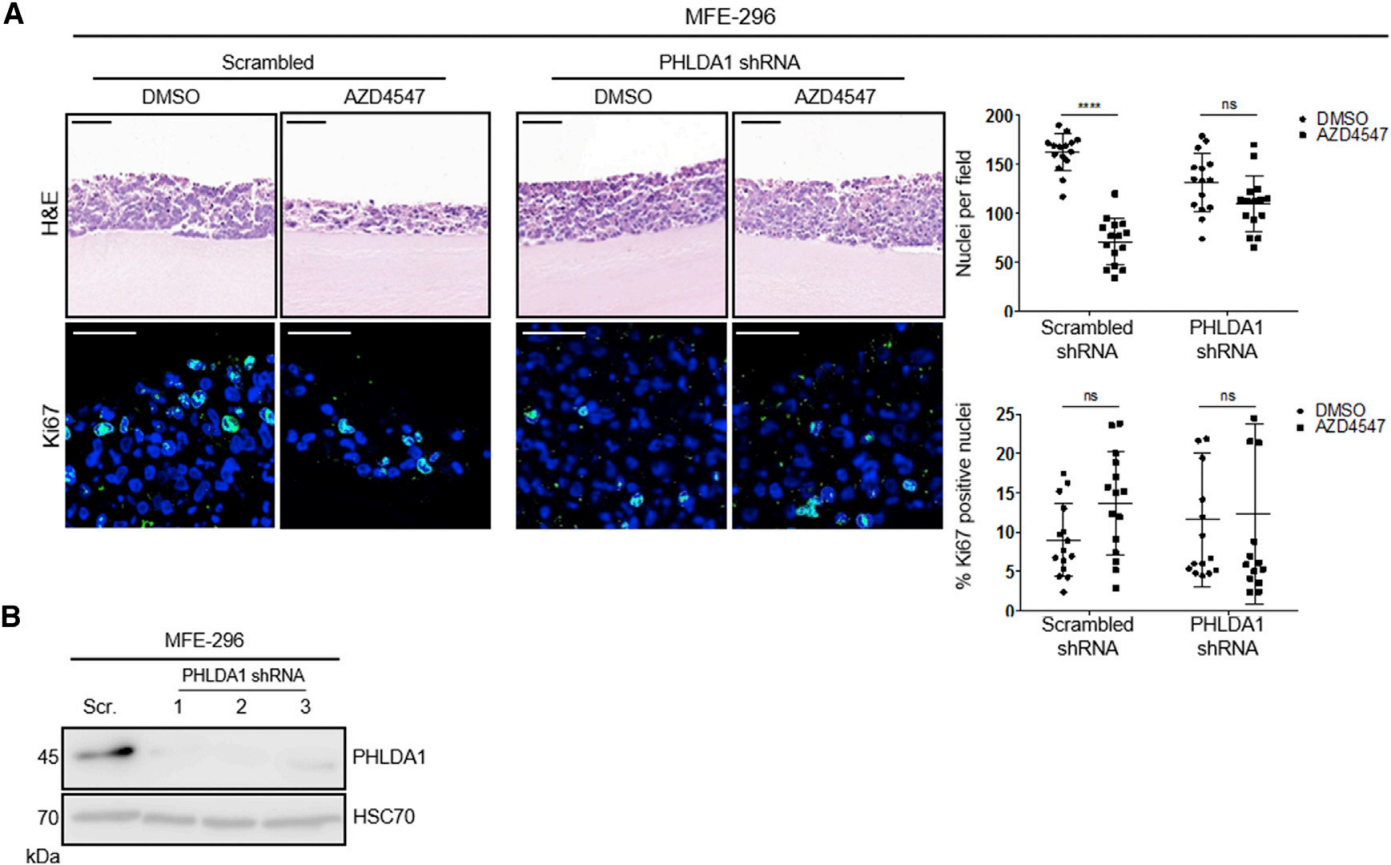
PHLDA1 Downregulation Confers *De Novo* Resistance to FGFR Inhibitors (A) Upper: H&E staining of MFE-296 cells expressing either scrambled or PHLDA1 shRNA, grown in mini-organotypic cultures for 14 days with or without 1 μM AZD4547. Lower: Ki67 staining with nuclei counterstained by DAPI. Images are representative of three independent experiments conducted with three distinct shRNA sequences. Right: quantitation of cell number and percentage of Ki67 positive nuclei. Data are presented as mean ± SEM. H&E image scale bar, 100 μm; Ki67 image scale bar, 50 μm. ^∗∗∗^p ≤ 0.001. H&E images are automatically spliced composites. (B) Western blot analysis of PHLDA1 levels in MFE-296 expressing either scrambled or PHLDA1 shRNA.

Having identified *PHLDA1* as a significantly downregulated gene in resistant cell populations, we examined whether PHLDA1 loss alone was sufficient to confer resistance in parental cell lines. We engineered four lentiviral short hairpin RNA (shRNA) constructs (three targeting PHLDA1 and one scrambled non-targeting control) and generated cell lines stably expressing each shRNA. After 14 days of culture, MFE-296 cells expressing scrambled shRNA sequences showed a marked reduction in cell number when exposed to 1 μM AZD4547, compared with DMSO controls (Figure 4A). Proliferation was unaffected between treated and untreated scrambled shRNA cells at 14 days of culture, indicating the scrambled controls had developed resistance akin to wild-type cells. Strikingly, this AZD4547 induced reduction in cell number was ameliorated significantly when PHLDA1 shRNA sequences were expressed, suggesting the acquisition of *de novo* resistance (Figure 4A). Effective knockdown of PHLDA1 at the protein level in MFE-296 cells was confirmed prior to embedding cells into mini-organotypic gels (Figure 4B).

### Recovery of PHLDA1 Expression Re-sensitizes Resistant Cells to FGFR Inhibitors

**Figure 5:**
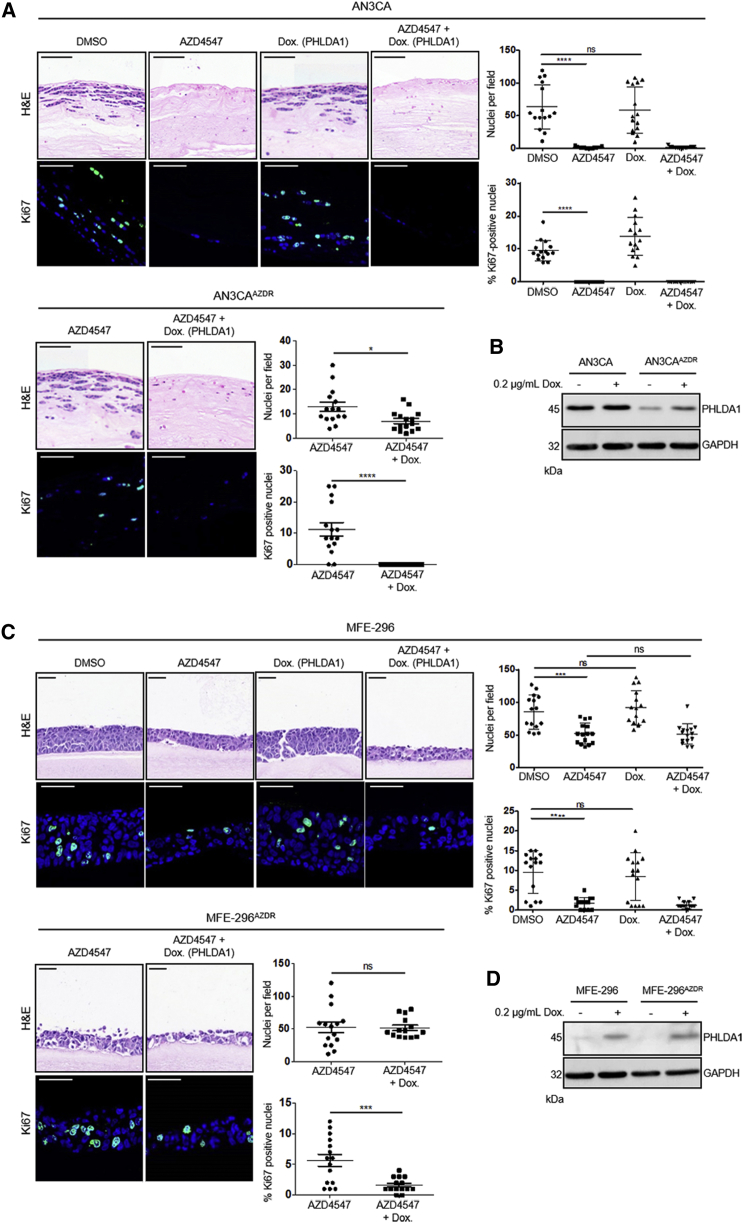
Recovery of PHLDA1 Expression Re-sensitizes Resistant Cells to FGFR Inhibitors (A and C) Upper: H&E staining of parental and AZD4547-resistant AN3CA (A) and MFE-296 cells (C) containing a doxycycline-inducible PHLDA1 expression construct. Cells were grown in mini-organotypic cultures for 7 days with or without 1 μM AZD4547 and 0.2 μg/mL doxycycline. Lower: Ki67 staining with nuclei counterstained by DAPI. Right: quantitation of cell number and percentage of Ki67 positive nuclei. Data are presented as mean ± SEM. Images are representative of at least three independent experiments. H&E image scale bar, 100 μm; Ki67 image scale bar, 50 μm. ^∗∗∗^p ≤ 0.001. H&E images are automatically spliced composites. (B and D) Western blot showing PHLDA1 levels in parental and resistant AN3CA cells (B) and MFE-296 cells (D) following doxycycline treatment. Data are representative of three independent experiments.

Having determined that PHLDA1 downregulation was associated with an ability to grow in the presence of FGFR inhibitor, we investigated whether rescuing the expression of PHLDA1 was sufficient to re-sensitize cells resistant to FGFR inhibitors. Full-length human PHLDA1 was cloned into a doxycycline inducible lentiviral construct and transduced into parental and AZD4547-resistant AN3CA and MFE-296 cells. As expected, parental AN3CA cells remained exquisitely sensitive to AZD4547, irrespective of PHLDA1 overexpression (Figure 5A, upper panel). However, AN3CA^AZDR^ cells, which showed similar growth in 1 μM AZD4547 as parental AN3CA cells did in the absence of drug, were completely re-sensitized to both FGFR inhibitors through the induction of PHLDA1 expression (Figure 5A, lower panel). Moreover, PHLDA1 induction in resistant cells in the absence of drug had no effect on cell growth (Figure S4). Efficacy of PHLDA1 induction was confirmed by western blot (Figure 5B), and the data were recapitulated in MFE-296 cells (Figures 5C and 5D), confirming that re-expression of PHLDA1, while having no effect on non-FGFR-inhibitor-treated cells, was sufficient to re-sensitize drug-resistant cells.

### PHLDA1 Mediates Resistance to RTK-Targeted Therapies in Breast Cancer

Having determined that PHLDA1 downregulation can underpin tyrosine kinase inhibitor resistance in endometrial cancer cell lines, we investigated whether this was a more global phenomenon in resistance to RTK-targeted therapy. We examined Human Epidermal Growth Factor Receptor 2 (HER2) positive breast cancers as not only is this RTK overexpressed in 25%–30% of breast cancers, but also 70% of patients develop resistance to the current frontline therapy, the HER2-targeted monoclonal antibody, trastuzumab (Moore et al., 2014, Slamon et al., 2001).

**Figure 6:**
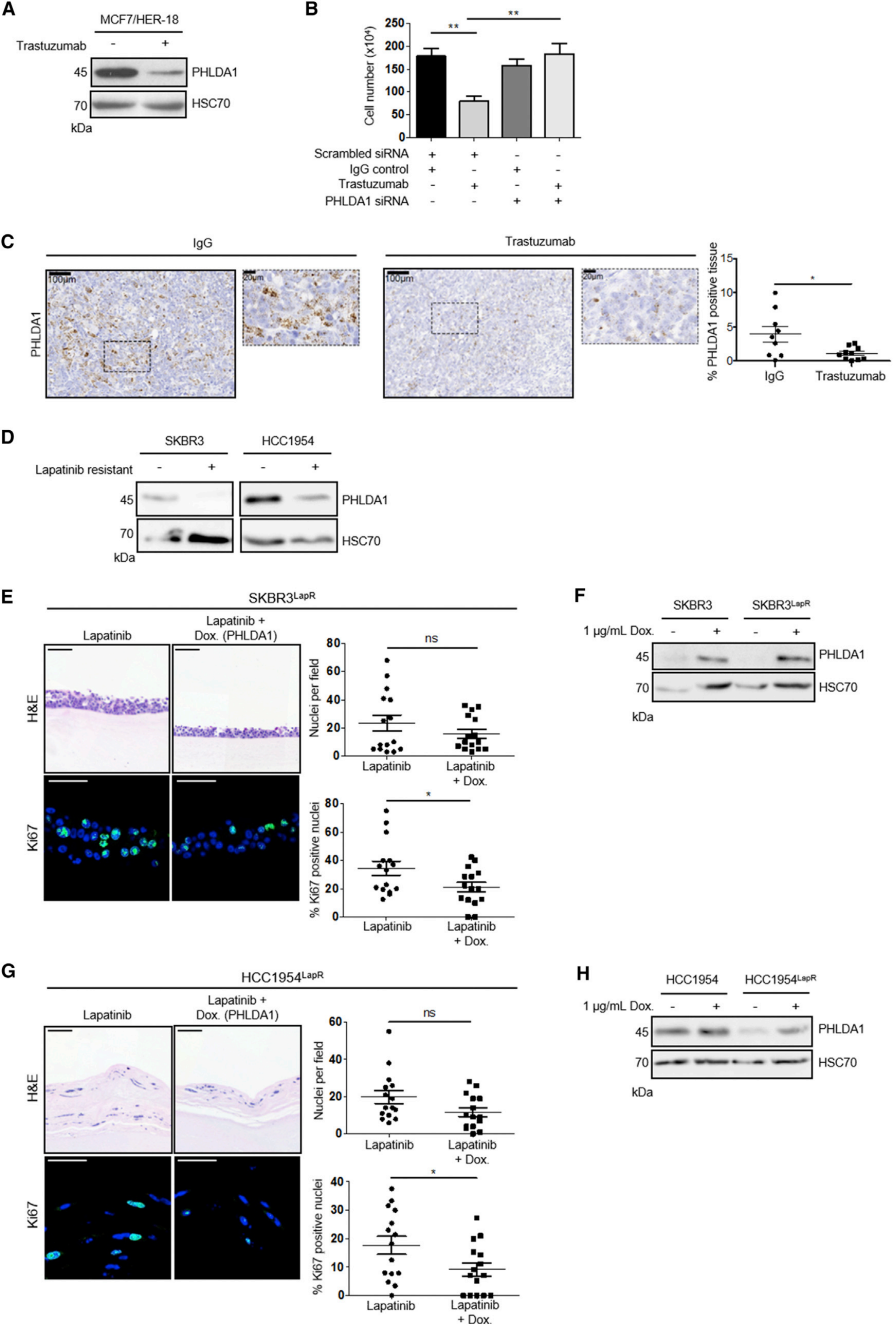
PHLDA1 Levels Regulate Sensitivity to Trastuzumab and Lapatinib Treatment (A) Western blot analysis of PHLDA1 levels in MCF7/HER2-18 cells cultured with 1 μM trastuzumab or IgG control for 72 hr. (B) MCF7/HER2-18 cell number following 3-day treatment with 1 μM trastuzumab preceded by 48-hr siRNA knockdown of PHLDA1 or scrambled control. (C) *In situ* hybridization for PHLDA1 expression in MCF7/HER2-18 xenograft tumors. Four-week-old tumors from mice treated with an IgG control showed strong PHLDA1 mRNA expression (brown), whereas treatment with trastuzumab resulted in significantly weaker staining, as shown in graph on right. Sections were counterstained with hematoxylin, and dotted boxes represent zoomed-in areas. Data are presented as mean ± SEM from at least eight mice for each condition. ^∗^p ≤ 0.05, ^∗∗^p ≤ 0.01, compared with IgG controls. (D) Western blot showing PHLDA1 levels in parental and lapatinib-resistant SKBR3 and HCC1954 cells treated with 2 μM lapatinib or DMSO control for 48 hr. (E and G). Upper: H&E staining of SKBR3^LapR^ (E) and HCC1954^LapR^ cells (G) containing a doxycycline-inducible PHLDA1 expression construct. Cells were grown in mini-organotypic cultures for 7 days with or without 2 μM lapatinib and 1 μg/mL doxycycline. Lower: Ki67 staining with nuclei counterstained by DAPI. Right: quantitation of cell number and Ki67-positive nuclei. Data are presented as mean ± SEM. Images are representative of at least three independent experiments. H&E image scale bar, 100 μm; Ki67 image scale bar, 50 μm. ^∗∗∗^p ≤ 0.001. H&E images are automatically spliced composites. (F and H) Western blot showing PHLDA1 levels in parental and resistant SKBR3 cells (F) and HCC1954 cells (H) following treatment with doxycycline.

PHLDA1 levels were reduced significantly in the HER2^+^ breast cancer cell line, MCF7/HER2-18 (Yu et al., 1996), following exposure to trastuzumab (Figure 6A). Similar to endometrial cancer cells, knockdown of PHLDA1 alone, by 48-hr treatment with small interfering RNA (siRNA), was sufficient to generate *de novo* resistance to trastuzumab (Figures 6B and S5A).

To examine this further in an *in vivo* setting, MCF7/HER2-18 cells were injected subcutaneously into immunodeficient mice, which subsequently received biweekly intraperitoneal injections of trastuzumab or immunoglobulin G (IgG) control for 4 weeks, as outlined previously (Moore et al., 2014). *In situ* hybridization for PHLDA1 showed significantly decreased mRNA levels upon trastuzumab treatment (Figure 6C), suggesting that downregulation of PHLDA1 might be a common response to RTK inhibition *in vitro* and *in vivo*.

In support of this, we determined the importance of PHLDA1 expression in parental and lapatinib-resistant populations of two further HER2^+^ breast cancer cell lines, SKBR3 and HCC1954. These resistant cell lines were generated through exposure to increasing concentrations of lapatinib and showed a marked reduction in sensitivity to lapatinib compared to parental counterparts (Figure S5B–S5E). In both lines, lapatinib-resistant populations showed a dramatic decrease in PHLDA1 protein levels (Figure 6D). Rescue of PHLDA1 in mini-organotypic models, using the same doxycycline inducible approach as above, led to a significant decrease in cell proliferation in lapatinib-resistant cell lines (Figures 6E–6H). Importantly, both SKBR3 and HCC1954 parental lines showed exquisite sensitivity to lapatinib treatment and were unaffected by induction of PHLDA1 above baseline levels (Figures S5F and S5G). Again, these data demonstrate that re-expression of PHLDA1 alone is sufficient to re-sensitize cells to RTK inhibition.

Since the development of RTKi resistance in our endometrial cancer model was dependent on a resurgence in Akt activity (Figure 2D), we examined whether the re-sensitization of resistant cells following PHLDA1 induction was mediated by a suppression of Akt signaling. Indeed, induction of PHLDA1 expression in both endometrial and breast cancer models resulted in a marked decrease in Akt phosphorylation (Figure S6A). This regulation of Akt activation was shown to be dependent on PHLDA1 PH domain function, since induction of mtPHLDA1 did not affect pAkt (Ser473) levels (Figure S6B). Importantly, HCC1954^LapR^ cells could not be re-sensitized to lapatinib treatment by expression of mtPHLDA1 (Figure S6C).

### Human Tumors Treated with RTK Inhibitors Show Lower PHLDA1 Expression Compared to Untreated Controls

To investigate whether PHLDA1 expression is downregulated in human tumors treated with RTK inhibitors, three Affymetrix datasets were obtained from the NCBI GEO database and the expression of PHLDA1 was compared between treated and untreated tumor samples. We examined locally advanced non-metastatic renal tumors treated with the PDGFR/VEGFR inhibitor sunitinib, metastatic breast cancers treated with docetaxel in combination with sunitinib, and metastatic ER^+^ breast tumors treated with tamoxifen in combination with the PDGF/VEGFR/Ras/Raf/MAPK inhibitor sorafenib (Figure S5H). In all cases, patients treated with an RTK inhibitor exhibited reduced PHLDA1, suggesting that PHLDA1 downregulation occurs in response to RTK inhibition in multiple tumor types.

## Discussion

Targeted therapies entering the clinic provide a powerful means to treat RTK-driven cancers. However, with this comes the challenge of cancers developing resistance, rendering the therapy ineffective (Holohan et al., 2013, Garraway and Jänne, 2012, Glickman and Sawyers, 2012). Identifying markers predictive of patient response to treatment, and methods to circumvent resistance, are thus of great importance for the continued use of targeted approaches in cancer treatment.

Here, we have utilized endometrial cancer cell lines harboring driver mutations in FGFR2 (Byron et al., 2008, Dutt et al., 2008, Pollock et al., 2007) as a platform to identify mechanisms of tyrosine kinase inhibitor (TKI) resistance. As FGFR mutations account for approximately 16% of endometrial cancers, they present an attractive target for therapy either as single agents or in combination with conventional chemotherapy (Byron et al., 2012, Konecny et al., 2013). However, due to a high prevalence of chemotherapy resistance in endometrial cancer (Chaudhry and Asselin, 2009), the potential impact of FGFR therapies is challenged. MFE-296 cells readily acquired resistance to the FGFR inhibitors PD173074 and AZD4547 following continued exposure in 2D and 3D cultures. AN3CA cells were consistently more sensitive to FGFR inhibition but nevertheless developed resistance to FGFR inhibitors through persistent exposure.

Acquisition of resistance to FGFR inhibitors has been reported previously (Packer et al., 2017), but the mechanistic basis of this remains to be elucidated. Through phosphoproteomic analysis of resistant cells, we identified a recovery in Akt-mediated signaling following FGFR inhibition. Recovery of the PI3K/Akt pathway has previously been suggested as a driver of resistance following RTK inhibition (Goltsov et al., 2011), and this is also true for FGFR-driven cancers (Singleton et al., 2015, Wang et al., 2017, Datta et al., 2017, Packer et al., 2017). Crucially, inhibition of FGFR and Akt signaling in our models was sufficient to prevent the acquired resistance to FGFR inhibition, demonstrating that recovery of Akt signaling confers resistance to FGFR inhibition.

PI3K/Akt signaling is frequently deregulated in cancer, making inhibition of this pathway an attractive approach (Foster et al., 2012). However, cancers are also capable of developing resistance to PI3K directed therapies, which are often mediated through the MEK pathway, another prominent driver of cell growth and survival (Hoeflich et al., 2009, Wee et al., 2009). Thus, combination treatments are a potential method to overcome resistance to a single agent. This approach has been demonstrated to be effective with combined treatments of PI3K and MEK-targeted therapeutics, but at the expense of severe dose-limiting toxicities (Shimizu et al., 2012). A more effective strategy will therefore arise from the identification, and therapeutic targeting, of novel resistance pathways that are not themselves critical for normal cell function.

To determine the changes in gene expression that underpinned the recovery in Akt signaling, we compared gene expression profiles of MFE-296 endometrial cancer cells, resistant to two distinct FGFR inhibitors, with that of parental cells. The most striking result from our microarray analysis was that, in both populations of resistant endometrial cancer cell lines, the Pleckstrin Homology-Like Domain-containing protein, PHLDA1, was the most strongly downregulated gene. PHLDA1 has been shown to negatively regulate Akt activation (Durbas et al., 2016, Li et al., 2014), and the concurrent downregulation of PHLDA1 and resurgence of Akt signaling observed in our model suggested a role for PHLDA1 loss in the development of TKI resistance. Indeed, our experiments showed that PHLDA1 downregulation is critical for resistance to FGFR inhibition; not only does PHLDA1 knockdown confer *de novo* resistance in parental cell lines, but re-expression in resistant lines is sufficient to re-sensitize cells to FGFR inhibition.

Despite the robust effect on resistance of manipulating PHLDA1 in our model systems, other genes identified in our microarray analysis may be involved in resistance. Indeed, DUSP6 downregulation has been implicated in resistance to EGFR-targeted therapy in lung cancer (Phuchareon et al., 2015). However, this resistance mechanism was driven through the reactivation of the ERK pathway despite the continued suppression of Akt, whereas in our system resurgence in Akt signaling is required for resistance.

Our data also suggest that PHLDA1 loss in resistance is not a response restricted to FGFR inhibition but may reflect a more global mechanism of resistance to RTK inhibition. PHLDA1 loss is able to confer resistance to trastuzumab in both *in vitro* and *in vivo* models of HER2^+^ breast cancer. Moreover, as with our endometrial cancer cell lines, re-expression of PHLDA1 re-sensitizes lapatinib-resistant HER2^+^ breast cancer cells. PHLDA1 loss has been observed in response to HER2-targeted therapy (Li et al., 2014), but here we demonstrate that loss of PHLDA1 alone is sufficient to account for this resistance. Mining publically available clinical datasets, we identified expression of PHLDA1 in breast and renal tumors from patients treated with PDGFR/VEGFR inhibitors, either alone or in combination with other therapies, was significantly lower than in tumors from patients treated with chemotherapy or hormone therapy alone, further supporting a broader role for PHLDA1 loss as a mechanism of resistance to kinase inhibitors.

**Figure 7:**
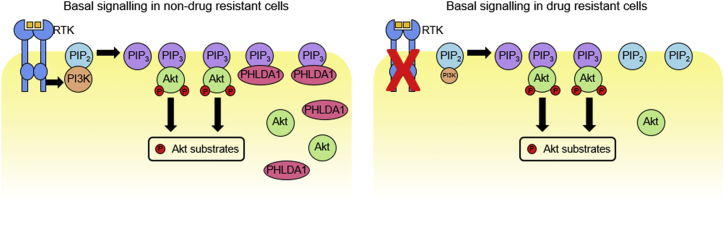
Model for PHLDA1 Silencing as a Mechanism of Acquired Drug Resistance In normal growth conditions (left), strong PI3K activity results in generation of ample PIP_3_ at the cell membrane. This enables the recruitment of Akt and PDK1 (not shown), resulting in Akt phosphorylation and subsequent activation. PHLDA1 can buffer this signaling by competing for free PIP_3_. When RTK activity is blocked by pharmacological inhibition (right), PI3K activity is reduced, leading to a reduction in free PIP_3_. This results in decreased Akt signaling, and reduced cell division/survival. Cells can establish resistance by silencing *PHLDA1* expression, thus removing the competition for free PIP_3_ binding. This would allow Akt signaling to recover, even in the absence of a strong RTK driver.

PHLDA1 was originally identified as a pro-apoptotic protein involved in T cell receptor activation-induced apoptosis (Frank et al., 1999, Park et al., 1996) and has since been shown to inhibit cell proliferation and invasion in a number of cell types (Bonatto et al., 2017, Johnson et al., 2011, Neef et al., 2002, Oberst et al., 2008, Gomes et al., 1999). The mechanistic basis for these cellular functions of PHLDA1 is largely unknown. The PHLDA1 PH domain shares high sequence homology with the PH domain of the related protein PHLDA3, which has been shown to compete with Akt for binding to PIP_3_ (Kawase et al., 2009). We have demonstrated that PHLDA1 expression is capable of suppressing Akt activity and also localizes to the plasma membrane in a PH-domain-dependent manner, suggesting that PHLDA1 can regulate Akt in a similar fashion to PHLDA3. Our data, together with these published findings, support a model where loss of PHLDA1 during sustained TKI treatment allows for sufficient Akt recruitment to residual PIP_3_ in order to maintain cell proliferation and survival (Figure 7).

It remains to be determined whether initial PHLDA1 expression in a tumor influences the response to treatment and whether this can be used to identify patients who are likely to develop resistance. The potential for PHLDA1 as a prognostic factor in cancer appears to be context specific, as high expression is associated with poor prognosis in ER^+^ breast cancers (Kastrati et al., 2014), and contributes to intestinal and pancreatic tumorigenesis (Oberg et al., 2004, Sakthianandeswaren et al., 2011), while low expression is a poor indicator in ER^–^ cancers (Johnson et al., 2011, Nagai et al., 2007), and is linked with cancer progression in melanoma (Neef et al., 2002) and gastric adenocarcinoma (Zhao et al., 2015).

We have identified PHLDA1 downregulation as a mechanism by which cancer cells can develop resistance to RTK-targeted therapy. Our data support a model whereby co-activation of downstream signaling pathways leads to drug resistance (Stommel et al., 2007). We propose that, by lowering PHLDA1 levels, the threshold level of PIP_3_ required for sufficient Akt activation is reduced, allowing the relatively weaker PI3K activity downstream of alternative RTKs to generate sufficient signaling to support cell proliferation. Thus, PHLDA1 may represent a useful biomarker to identify patients who will develop resistance to cancer therapeutics, and targeting PHLDA1 regulation presents an attractive prospect for preventing drug resistance in cancer patients.

## Experimental Procedures

Further details and an outline of resources used in this work can be found in Supplemental Experimental Procedures.

### 3D Organotypic Model

Organotypic cultures were prepared following a modified version of a previously published protocol (Chioni and Grose, 2012). Briefly, collagen/Matrigel gels containing 5 × 10^5^ human foreskin fibroblasts (HFF2) cells/mL were overlaid with 1 × 10^6^ cancer cells/mL and raised to an air-liquid interface upon a nylon-membrane covered metal grid in a 6-well plate. At the relevant time point, gels were fixed in 10% neutral buffered formalin, bisected and dehydrated in 70% ethanol before paraffin embedding.

The mini-organotypic model was modified from a previously described protocol (Coleman et al., 2014). A collagen/Matrigel gel containing 6.25 × 10^4^ HFF2 cells was prepared in a 24-well plate transwell insert (Corning, 3412) and overlaid with 1.25 × 10^5^ cancer cells. Cells were left to adhere to the gel then subsequently cultured at the air-liquid interface. Gels were formalin fixed, paraffin embedded and sectioned as described above.

### Western Blotting

Cell lysates were prepared using RIPA buffer (Millipore) supplemented with protease (Millipore) and phosphatase (Millipore) inhibitor cocktails. Denatured proteins (20–40 μg) were separated by electrophoresis on 4%–12% Bis-Tris gels (NuPAGE Novex; Invitrogen). Proteins were subsequently transferred onto nitrocellulose membranes, blocked with 5% milk, and incubated with primary antibody, diluted 1:1,000 in 3% BSA/PBS. All antibodies were rabbit polyclonal unless otherwise stated: anti-Akt (Cell Signaling Technology, 9272S), anti-ERK (Millipore, 06-182), anti-FGFR2 (Santa Cruz, sc-122), anti-FRS2 (Santa Cruz, sc-8318), anti-HSC70 (mouse monoclonal; Santa Cruz, sc-7298), anti-p-Akt (Ser473) (Cell Signaling Technology, 9271S), anti-p-ERK (Thr202/Tyr204) (Cell Signaling Technology, 9101S), anti-p-FRS2 (Cell Signaling Technology, 3861), anti-PHLDA1 (Abcam, ab133654). Membranes were then incubated with a species appropriate horseradish peroxidase (HRP)-conjugated secondary antibody (DAKO) before bands were visualized using an enhanced chemiluminescence detection kit (GE Healthcare).

### Mass Spectrometry

MFE-296 cells were plated at 7 × 10^5^ cells per 10-cm dish and either left untreated or cultured with DMSO as a vehicle control, or 1 μM PD173074. Cells were lysed at 1, 7, or 14 days. Cell lysis, digestion, solid-phase extraction, TiO_2_ Metal Oxide Affinity Chromatography, Nanoflow-liquid chromatography tandem mass spectrometry, and identification and quantification of phosphopeptides were performed as previously described (Wilkes et al., 2015).

### Gene Expression Microarray

Microarray gene expression analysis of cDNA from two biological replicates of each cell line was performed using the Illumina HT12v platform at Barts Genome Centre. Each sample was run on the array in duplicate. The resulting data were analyzed using Illumina Genome Studio software. Within the software, data quality control, filtering, and normalization were performed across samples. Differential expression analyses between the two biological groups were further conducted. Significantly differentially expressed genes were identified based on the adjusted p value <0.05 using Bonferroni correction. The gene expression microarray data have been deposited to GEO using the accession number GSE81169.

### Tumor Xenografts

Female SCID mice were subcutaneously injected with MCF7/HER2-18 cells at 6–8 weeks of age (Moore et al., 2014). Mice were randomized into treatment groups based on tumor volume (n ≥ 3/treatment) and administered biweekly intraperitoneal injections (10 mg/kg in 200 μL of PBS) of human IgG or trastuzumab for 4 weeks. Tumors were harvested and fixed as described previously, and 5-μm wax sections were processed for *in situ* hybridization. All mouse experiments followed Home Office Guidelines determined by the Animals (Scientific Procedures) Act 1986.

## Data and Software Availability

The accession number for all microarray datasets reported in this paper is GEO: GSE81169. The dataset identifier for the mass spectrometry proteomics is ProteomeXchange Consortium via the PRIDE (Vizcaíno et al., 2016) partner repository: PXD008859.

## Supplemental Information

Document S1. Supplemental Experimental Procedures and Figures S1–S6

Document S2. Article plus Supplemental Information
