# PHLDA1 Mediates Drug Resistance in Receptor Tyrosine Kinase-Driven Cancer

**DOI:** 10.1016/j.celrep.2026.117552

**Published:** 2026-06-03

**Authors:** Abbie E. Fearon, Edward P. Carter, Natasha S. Clayton, Edmund H. Wilkes, Ann-Marie Baker, Ekaterina Kapitonova, Bakhouche A. Bakhouche, Yasmine Tanner, Jun Wang, Emanuela Gadaleta, Claude Chelala, Kate M. Moore, John F. Marshall, Juliette Chupin, Peter Schmid, J. Louise Jones, Michelle Lockley, Pedro R. Cutillas, Richard P. Grose

## Main text

(Cell Reports *22*, 2469–2481; February 27, 2018)

In Figure 5A, the image for AZD4547 H&E staining was inadvertently duplicated, appearing also as the representative image for H&E staining in PHLDA1. Although the paper cannot be directly updated because of the amount of time that has passed since its initial publication, the revised figure can be seen below. The authors apologize for the error, which was unintentional and occurred during figure preparation.

**Figure 5:**
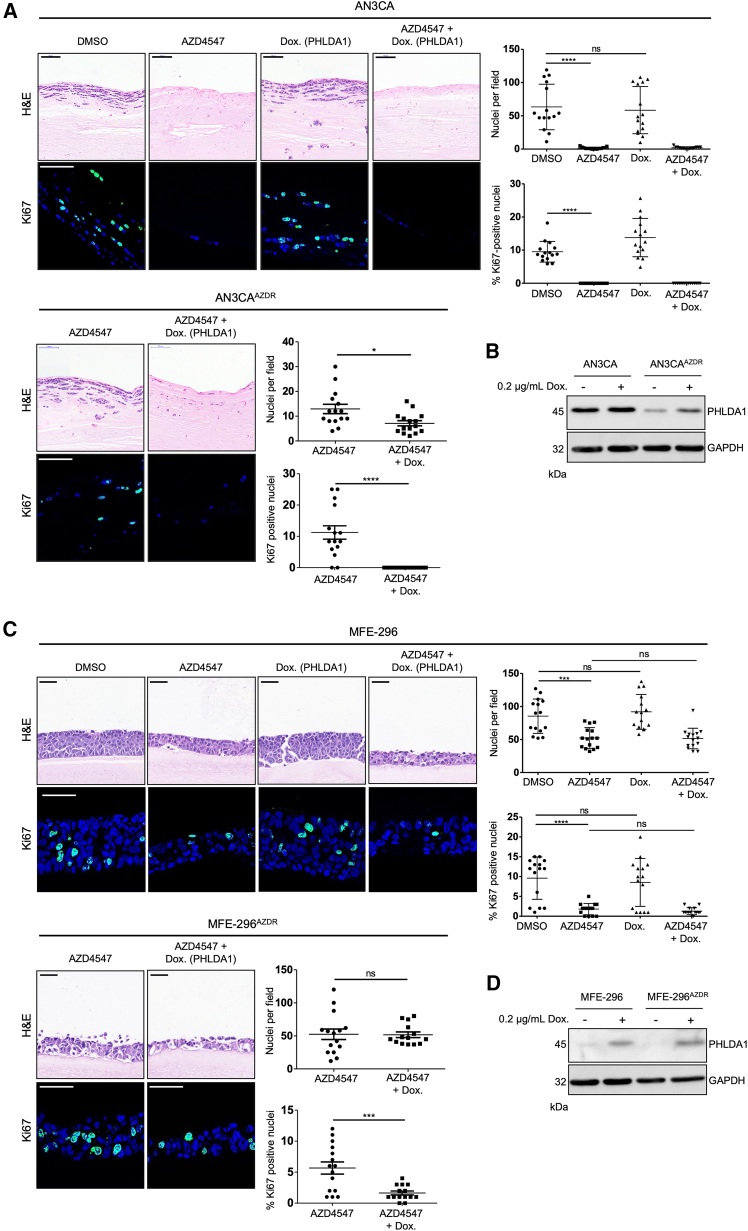
Recovery of PHLDA1 Expression Re-sensitizes Resistant Cells to FGFR Inhibitors (corrected)

