# Supplementary material for: PHLDA1 Mediates Drug Resistance in Receptor Tyrosine Kinase-Driven Cancer
Source: Cell Rep. 2018 Feb 28;22(9):2469–81. doi: 10.1016/j.celrep.2018.02.028 (PMC5848852; doi:10.1016/j.celrep.2018.02.028)
Supplement: Document S1. Supplemental Experimental Procedures and Figures S1–S6 [file mmc1.pdf]

Cell Reports, Volume *22*

Supplemental Information

PHLDA1 Mediates Drug Resistance in Receptor Tyrosine Kinase-Driven Cancer

Abbie E. Fearon, Edward P. Carter, Natasha S. Clayton, Edmund H. Wilkes, Ann-Marie Baker, Ekaterina Kapitonova, Bakhouche A. Bakhouche, Yasmine Tanner, Jun Wang, Emanuela Gadaleta, Claude Chelala, Kate M. Moore, John F. Marshall, Juliette Chupin, Peter Schmid, J. Louise Jones, Michelle Lockley, Pedro R. Cutillas, and Richard P. Grose

Supplementary Experimental Procedures

Cell culture

Cells were obtained from the following suppliers: MFE-296 cells (Health Protection Agency, HPA); AN3CA, SKBR3, HCC1954 and HFF2 cells (American Type Culture Collection, ATCC); Ishikawa cells (Sigma-Aldrich); MCF7/HER2-18 cells were a kind gift of Prof Mien-Chie Hung (Yu et al., 1996). All cell lines were cultured according to the recommended guidelines of the supplier.

2D Proliferation assay

Cells were seeded into 96-well plates at a density of 1000 cells/well. After 16 hours, cells were treated as indicated and transferred to an IncuCyte® ZOOM imaging system maintained at 37°C, 5% CO_2. Each well was imaged at four positions every 2 hours using a Nikon 10x objective. Three technical replicates were performed for each condition and each experiment was performed on three separate occasions. Cell confluence was analysed using IncuCyte ZOOM software (version 20151.2.5599). For each time point, the mean confluence of each well was normalised to the mean confluence at 0 h of treatment. Growth curves were generated using mean fold change in confluence across three independent experiments.

Immunofluorescence and immunohistochemistry

Cells expressing GFP-PHLDA1, GFP-mtPHLDA1 and GFP-PH-Akt were cultured on glass coverslips and fixed in 10% neutral buffered formalin. Cells were then permeabilised with 0.05% saponin and blocked with 6% BSA. Coverslips were incubated with 1 µM Alexa Fluor 546 phalloidin and 1 µg/ml DAPI before being mounted onto microscope slides using Mowiol. Fluorescent images were acquired using a Carl Zeiss LSM710 confocal microscope.

Ki67 staining: Paraffin sections (4 µm) of organotypic cultures were dewaxed and blocked with 6% BSA/PBS following antigen retrieval in 10 mM sodium citrate buffer, pH 6.0. Sections were subsequently incubated with rabbit anti-Ki67 antibody (Abcam, ab15580) diluted 1:200 in 6% BSA/PBS. Sections were then incubated with a FITC-conjugated goat anti-rabbit antibody (Invitrogen, A11008) diluted 1:200 in 6% BSA/PBS. Slides were mounted using aqueous mounting medium supplemented with DAPI. ImageJ software was used to quantify DAPI and Ki67 staining from six random fields of view per section.

siRNA knockdown of PHLDA1

Cells were transfected with 10 nM of either a pool of four PHLDA1 siRNA oligonucleotides (Dharmacon, M-01238901) or a pool of non-targeting siRNA (Dharmacon, D-001810-10-20) using INTERFERin (Polyplus) transfection reagent following manufacturer's guidelines.

Lentiviral vectors and infection

shRNA lentiviral particles were generated by co-transfecting HEK293T cells with the packaging plasmids pMD2.G (Addgene #12259) and pCMVR8.2 (Addgene #12263) and either a pLKO.1 shRNA control (Addene #1864) or PHLDA1 shRNA (Sigma Mission) vector plasmid using Fugene HD transfection reagent (Promega)

following manufacturers guidelines. Viral-containing supernatant was collected 48 hours post transfection and applied directly to cultures of either MFE-296 or AN3CA cells. Infected cells were selected through culture in the presence of 1 µg/mL puromycin for 14 days prior to use. shRNA sequences are; shRNA control – CCTAAGGTTAAGTCGCCCTCGCTCGAGCGAGGGCGACTTAACCTTAGG. shRNA 1 (Sigma Mission #TRCN0000150307) – CCGGCCTAATCCGTAGTAATTCCTACTCGAGTAGGAATTACTACGGATTAGGTTTTTG. shRNA 2 (Sigma Mission #TRCN0000150983) – CCGGCAGATCAAGTAGTTTGGACATCTCGAGATGTCCAAACTACTTGATCTGTTTTTG. shRNA 3 (Sigma Mission # TRCN0000152275) – CCGGCGAGCACATTTCTATTGTCTTCTCGAGAAGACAATAGAAATGTGCTCGTT

To generate a doxycycline inducible PHLDA1 expression construct, the full coding sequence of human *PHLDA1* (NM_007350.3) was cloned into pDONR™221 (Invitrogen) and then transferred to pINDUCER21 (ORF-EG) (Addgene #46948) using Gateway™ technology (Invitrogen). Lentiviral production and transduction was performed as described above.

In situ hybridisation

In situ hybridisation for *PHLDA1* mRNA expression was performed on FFPE tissue sections using the RNAscope 2.0 High Definition assay (Advanced Cell Diagnostics, Hayward, CA) as previously described (Baker et al., 2015). RNAscope probes used were *PHLDA1* (NM_007350.3, region 168-1460, catalogue number 440831), *PPIB* (positive control probe, NM_000937.4, region 139-989, catalogue number 313901) and *dapB* (negative control probe, EF191515, region 414-862, catalogue number 310043).

Site-directed mutagenesis

To generate mtPHLDA1, nucleotides encoding amino acids 152-159 and 167-171 were deleted from wild type PHLDA1 cDNA (NM_007350.3) using Quikchange Multi Site-Directed Mutagenesis Kit (Agilent Technologies, 200514). Following Sanger sequencing, mtPHLDA1 was then cloned into pgLAP1 (Addgene, #19702) to generate an N-terminal GFP-fusion construct. mtPHLDA1 was also cloned into pInducer21 to generate a doxycycline-inducible expression construct.

Analysis of PHLDA1 expression in clinical samples

Three Affymetrix datasets (GSE68629, GSE40837, and GSE54323), with cancer samples treated with RTK inhibitors, were downloaded from the NCBI GEO database (Barrett et al., 2013). All analyses were performed in R (v3.2.2), using Bioconductor and associated packages (https://www.bioconductor.org/). After the application of stringent quality control criteria (Asare et al., 2009), the data were quantile normalised, using the Robust Multi-array Average algorithm. mRNA profiles representing each biological condition were then investigated at both the probe-level and the gene-level. The expression profiles of probes representing the PHLDA1 gene (217996_at, 217997_at, 217999_s_at, 218000_s_at, 225842_at, 217998_at) were collapsed for each patient, with the mean value used to represent the gene expression summary. Gene-level expression was plotted against treatment groups in the data.

Supplementary References

Asare, A. L., Gao, Z., Carey, V. J., Wang, R. & Seyfert-Margolis, V. (2009). Power enhancement via multivariate outlier testing with gene expression arrays. *Bioinformatics,* 25, 48-53.

Baker, A. M., Graham, T. A., Elia, G., Wright, N. A. & Rodriguez-Justo, M. (2015). Characterization of LGR5 stem cells in colorectal adenomas and carcinomas. *Sci Rep,* 5, 8654.

Barrett, T., Wilhite, S. E., Ledoux, P., Evangelista, C., Kim, I. F., Tomashevsky, M., Marshall, K. A., Phillippy, K. H., Sherman, P. M., Holko, M., Yefanov, A., Lee, H., Zhang, N., Robertson, C. L., Serova, N., Davis, S. & Soboleva, A. (2013). NCBI GEO: archive for functional genomics data sets--update. *Nucleic Acids Res,* 41, D991-995.

Yu, D., Liu, B., Tan, M., Li, J., Wang, S. S. & Hung, M. C. (1996). Overexpression of c-erbB-2/neu in breast cancer cells confers increased resistance to Taxol via mdr-1-independent mechanisms. *Oncogene,* 13, 1359-1365.

Supplementary Figures

Supplementary Figure 1

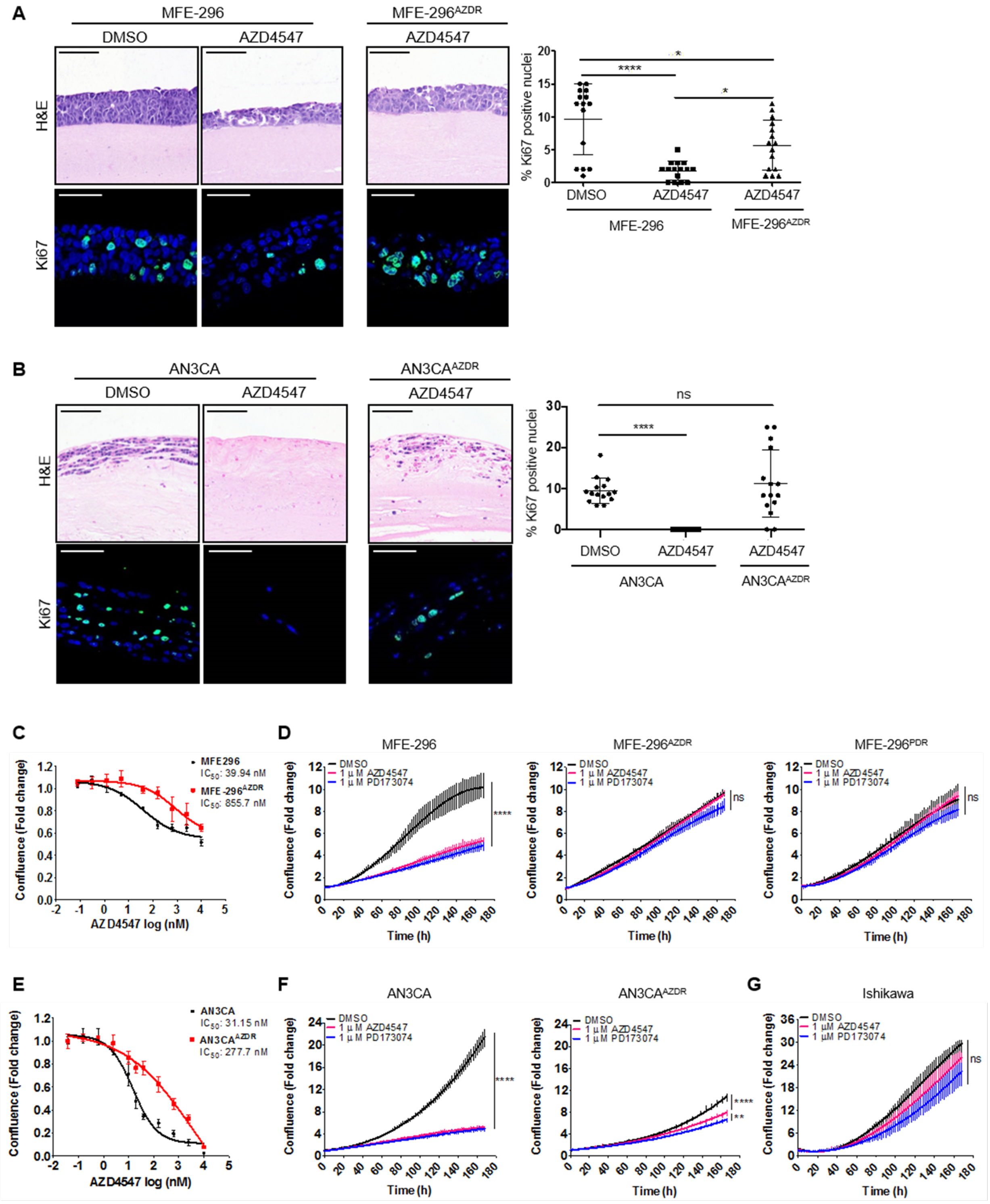

Supplementary Figure 1. (A and B) H&E staining (upper) and Ki67 staining (lower) of mini-organotypic cultures of parental or AZD4547 resistant MFE-296 (A) or AN3CA (B) cells cultured for 7 days with or without 1 µM AZD4547. Right: quantitation of Ki67-positive nuclei. Data presented as mean ±SEM. Images representative of at least three independent experiments. H&E image scale bar = 100 µm, Ki67 image scale bar = 50 µm. *** P≤0.001, compared with DMSO controls. (C-G) Effect of FGFR inhibitors on the growth of parental and resistant MFE-296 cells (C and D), AN3CA cells (E and F) and Ishikawa cells (G) as assessed by cell confluence data generated by IncuCyte® ZOOM system. H&E images are automatically spliced composites. Data presented as mean fold change in confluence from three independent experiments. *** P≤0.001.

Supplementary Figure 2

A

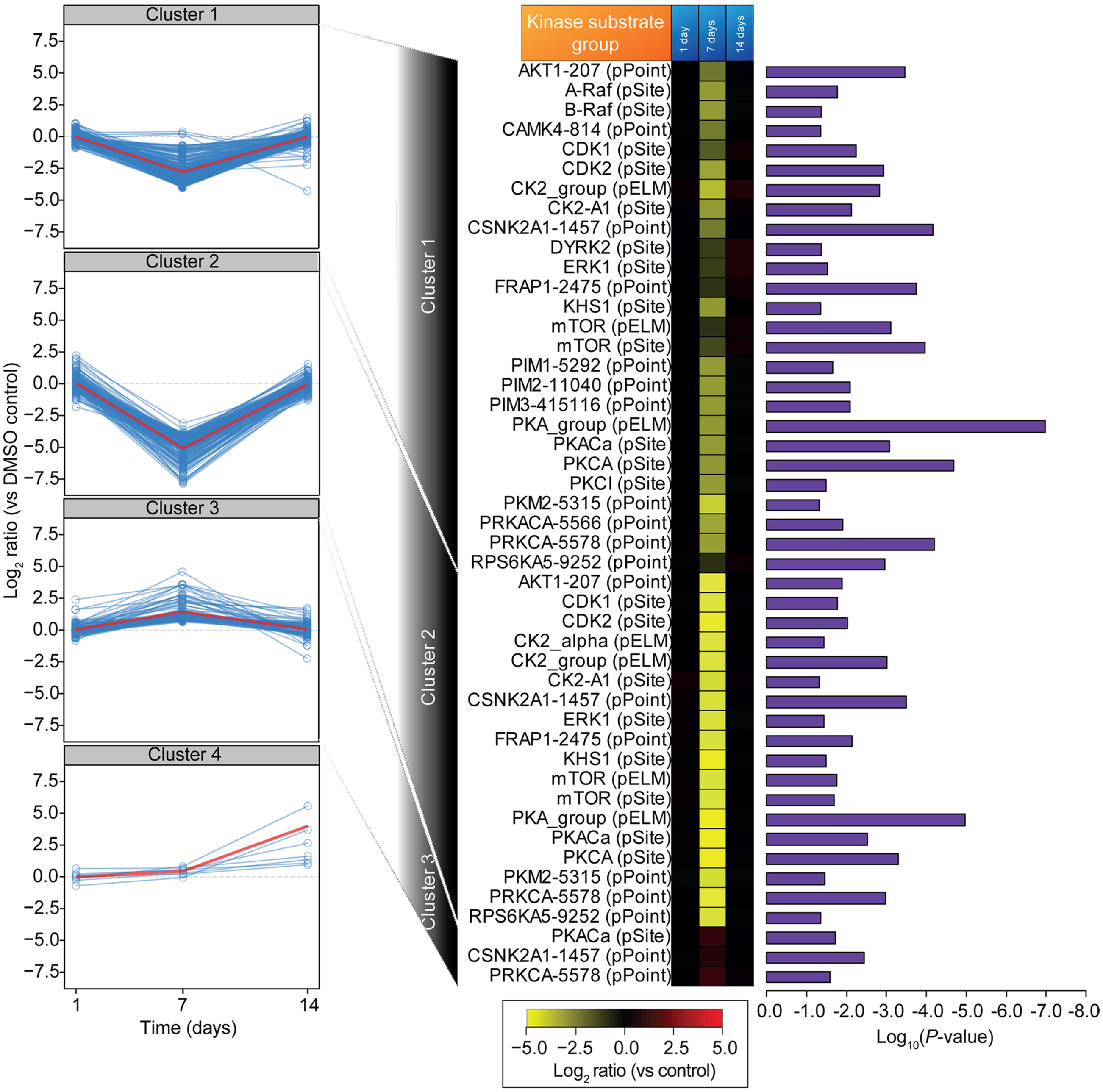

B

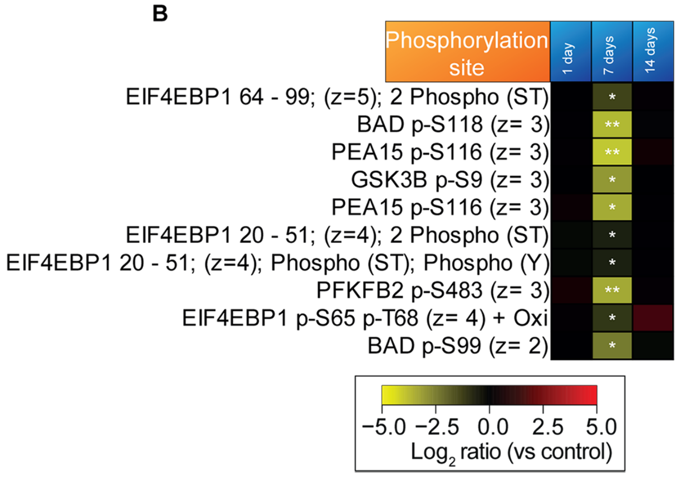

Supplementary Figure 2. Phosphoproteomic analysis of FGFR-inhibitor resistance acquisition in MFE-296 cells. (A) MS identified 6706 unique phospho-peptides in total across all samples. Of these, 525 were significantly up- or down-regulated in the PD173074 treated samples compared to the DMSO control for at least one time point, and were grouped according to their phosphorylation pattern, using unsupervised clustering (clusters 1-4; left panel). The resulting phosphopeptides were analysed using KSEA and grouped in a heatmap according to their upstream kinases (middle panel). P values of each group are shown as bars (right panel). pPoint, pSite and pELM in the heatmap represent the database employed by KSEA to cluster substrates into their kinase groups (phosphoPoint, phosphoSite and phospho.ELM respectively). Blue lines in the clusters represent individual phosphopeptides; the red lines represent the line of best fit. (B) Heatmap of phosphopeptides downstream of AKT which were significantly down regulated at seven days PD173074 treatment, compared to the DMSO control. z indicates number of potential phosphorylation sites identified on each peptide; 2 phospho indicates two phosphorylation sites were identified on the proceeding residues (S, serine; T, threonine; Y, tyrosine); pS118 etc indicates phosphorylation on S or T at the residue indicated by the number; Oxi indicates the phosphopeptide was oxidised; numbers preceding protein name indicate phosphopeptide length. Data represent average of two technical replicates of two biological replicates, i.e. each replicate was run through the MS twice. *, $P \leq 0.05$, **, $P \leq 0.01$.

Supplementary Figure 3

A

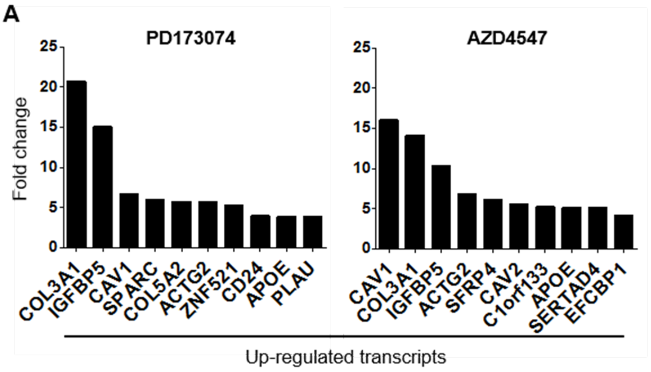

B

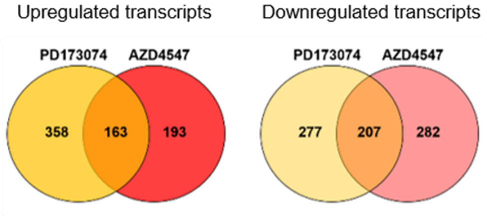

C

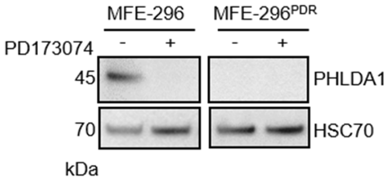

Supplementary Figure 3. (A) Top ten up-regulated genes in MFE-296^{PDR} (left) and MFE-296^{AZDR} (right) cells compared to parental controls, identified by microarray analysis. (B) Venn diagram showing the number of gene transcripts that were commonly upregulated (left) or downregulated (right) in MFE-296^{PDR} and MFE-296^{AZDR} cells. (C) Western blot showing down-regulation of PHLDA1 in MFE-296 cells following treatment with 1 μM PD173074 and persistent down-regulation of PHLDA1 in MFE-296^{PDR} cells following removal of 1 μM PD173074 for 24 hours.

Supplementary Figure 4

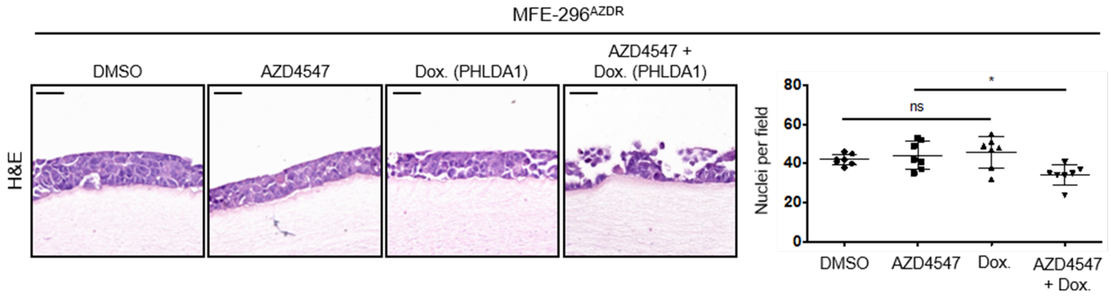

Supplementary Figure 4. Left: H&E staining of MFE-296^{AZDR} cells containing a doxycycline-inducible PHLDA1 expression construct. Cells were grown in mini-organotypic cultures for 7 days with or without 1 µM AZD4547 and 0.2 µg/mL doxycycline. Scale bar = 100 µm. Right: quantitation of cell number as determined by DAPI positive nuclei. Data points represent cell number per field of view. H&E images are automatically spliced composites. *** P≤0.001.

Supplementary Figure 5

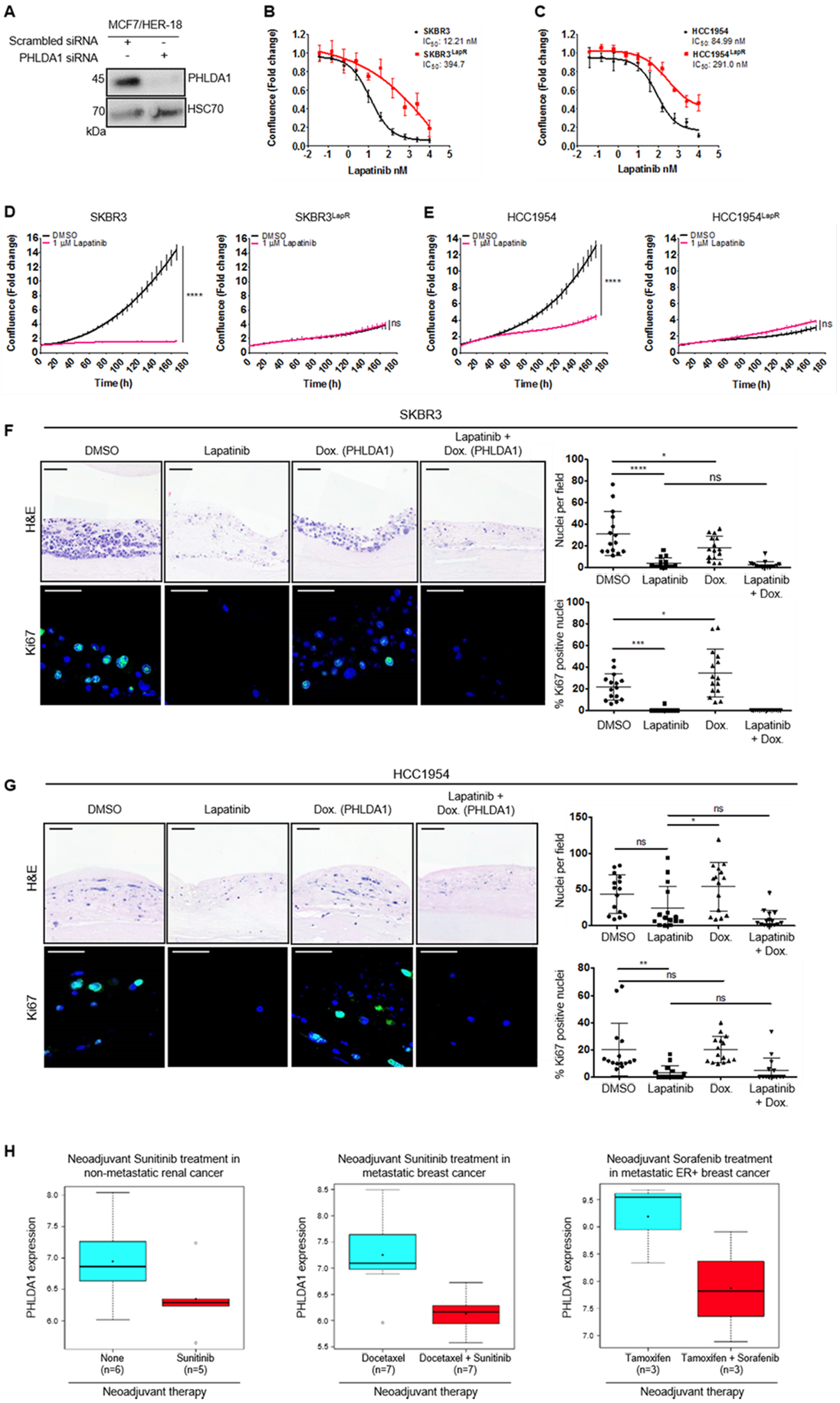

Supplementary Figure 5: (A) Western blot analysis of PHLDA1 levels in in MCF7/HER2-18 cells cultured with either scrambled control or PHLDA1 targeted siRNA for 48 hours. (B-E) Effect of lapatinib on the growth of parental and lapatinib resistant SKBR3 cells (B and D) and HCC1954 cells (C and E) as assessed by cell confluence data generated by IncuCyte® ZOOM system. Data presented as mean fold change in confluence from three independent experiments. *** P≤0.001. (F and G). Upper: H&E staining of parental SKBR3 (F) and HCC1954 cells (G) grown in mini-organotypic cultures for 7 days with or without 2 μM lapatinib and 1 μg/mL doxycycline to induce PHLDA1 expression. Lower: Ki67 staining with nuclei counterstained by DAPI. Right: Quantitation of DAPI positive nuclei and Ki67 positive nuclei. H&E images are automatically spliced composites. Data presented as mean ±SEM. Images representative of at least three independent experiments. H&E image scale bar = 100 μm, Ki67 image scale bar = 50 μm. *** P≤0.001. (H) Analysis of *PHLDA1* gene expression in human cancers treated with receptor tyrosine kinase inhibitors. Analysis was performed using three Affymetrix datasets (GSE68629, GSE40837 and GSE54323) downloaded from the NCBI GEO database. GSE68629: locally advanced non-metastatic renal tumours from patients treated with the PDGFR/VEGFR inhibitor sunitinib. GSE40837: Metastatic ER+ breast cancer tissue from patients treated with Tamoxifen in combination with the PDGF/VEGFR/Ras/Raf/MAPK inhibitor sorafenib. GSE54323: Metastatic breast cancer tissue from patients treated with Docetaxel in combination with the PDGFR/VEGFR inhibitor sunitinib.

Supplementary Figure 6

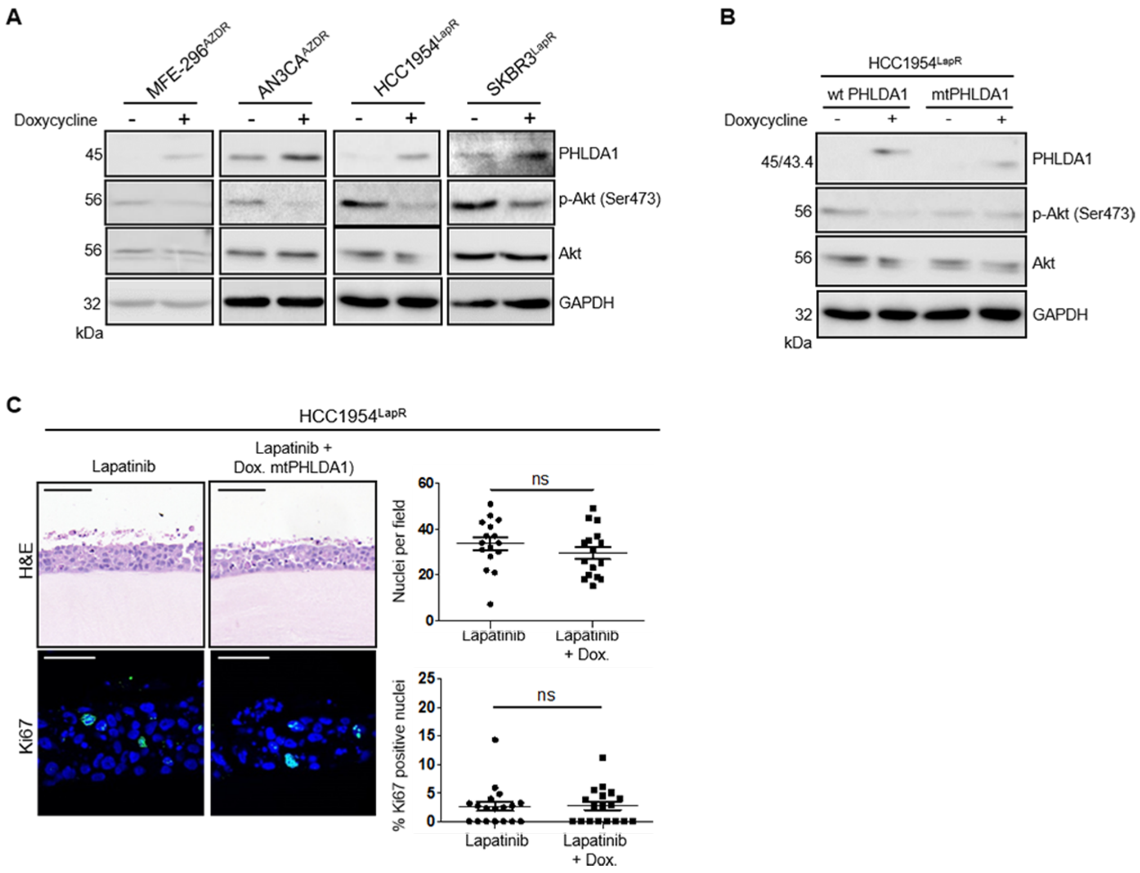

Supplementary Figure 6. (A) Western blot showing reduced p-Akt (Ser473) levels following induction of PHLDA1 expression in RTKi-resistant cells harbouring a doxycycline-inducible PHLDA1 expression construct. AZD4547 resistant MFE-296 and AN3CA cells, and lapatinib resistant HCC1954 and SKBR3 cells were treated with 2 µg/mL doxycycline for 48 hours. (B) Western blot showing induction of mtPHLDA1 expression (predicted molecular weight = 43.4 kDa) following treatment with 1 µg/mL doxycycline for 48 hours. mtPHLDA1 lacks a functional PH domain and expression of this mutant does not cause a decrease in p-Akt (Ser473). (C) Upper panels: H&E staining of $HCC1954^{LapR}$ cells containing a doxycycline-inducible mutant PHLDA1 (mtPHLDA1) expression construct. Cells were grown in mini-organotypic cultures for 7 days with or without 1 µM lapatinib and 1 µg/mL doxycycline to induce mtPHLDA1 expression. Scale bar = 100 µm. Lower panels: green Ki67 staining with nuclei counterstained by DAPI in blue. Scale bar = 50 µm. Right panels: quantitation of cell number and percentage of Ki67 positive nuclei. H&E images are automatically spliced composites. Data presented as mean ±SEM. Images representative of at least three independent experiments. *** $P \leq 0.001$.
